# Characteristics of attentional bias in adolescents with major depressive disorders: differentiating the impact of anxious distress specifier

**DOI:** 10.3389/fpsyt.2024.1352971

**Published:** 2024-03-18

**Authors:** Rong Yang, Hongyu Zheng, Xiaomei Cao, Daming Mo, Mengting Li, Wenyuan Liu, Hui Zhong

**Affiliations:** ^1^ Department of Child and Adolescents, Hefei Fourth People’s Hospital, Hefei, China; ^2^ School of Mental Health and Psychological Sciences, Anhui Medical University, Hefei, China; ^3^ Department of Child and Adolescents, Affiliated Psychological Hospital of Anhui Medical University, Hefei, China; ^4^ Department of Child and Adolescents, Anhui Mental Health Center, Hefei, China

**Keywords:** attentional bias, adolescent, major depressive disorder, anxious distress specifier, mood

## Abstract

**Background:**

No consistent conclusion has been reached regarding the attentional bias characteristics of adolescents with major depressive disorders (MDD), and unexamined co-occurring anxiety distress may contribute to this inconsistency.

**Methods:**

We enrolled 50 MDD adolescents with anxiety distress, 47 MDD adolescents without anxiety distress and 48 healthy adolescents. We measured attentional bias using a point-probe paradigm during a negative-neutral emotional face task. Reaction time, correct response rate and attentional bias value were measured.

**Results:**

MDD adolescents did not show a negative attentional bias; MDD adolescents with anxiety distress exhibited longer reaction time for negative and neutral stimuli, lower correct response rate for negative stimuli. Hamilton Anxiety Scale scores were positively correlated with reaction time, negatively correlated with correct response rate, and not significantly correlated with attentional bias value.

**Limitations:**

The cross-sectional design hinders causal attribution, and positive emotional faces were not included in our paradigm.

**Conclusion:**

Negative attentional bias is not a stable cognitive trait in adolescents with MDD, and avoidance or difficulty in disengaging attention from negative emotional stimuli may be the attentional bias characteristic of MDD adolescents with anxiety distress.

## 1 Introduction

Major Depressive Disorders (MDD) are a class of mental disorders characterized by the presence of significant and persistent low mood. In 2008, the World Health Organization listed MDD as the third leading contributor to the global burden of disease and predicted that by 2030, MDD will become the primary contributor (1, 2). The lifetime prevalence of adolescent MDD has been reported to be as high as 11.4% (3). Due to factors such as poor family environment, tense parent-child relationships, and high pressure to succeed in education, the incidence of depressive disorders among adolescents in China is increasing year by year. According to the *China National Mental Health Development Report* (2019-2020), the prevalence of depressive disorders among adolescents in China is 24.6%, with 7.4% classified as major depression. While MDD itself seriously affects academic performance and quality of life in adolescents, anxious distress is a prominent feature of MDD; moreover, high levels of anxiety have been associated with an increased risk of suicide, longer disease duration, and the likelihood of ineffective treatment (4, 5). The condition has been labeled ‘anxious distress specifier (ADS) for major depressive disorder’ in the U.S. version of the Diagnostic and Statistical Manual of Mental Disorders, 5th Edition (DSM-V) (6, 7). Patients with major depressive disorder with ADS reported more severe depressive symptoms, a higher number of hospitalizations, elevated rates of suicidal ideation, increased illness severity, greater work impairment, diminished quality of life, and self-perceived cognitive impairment (8, 9). Currently, several groups have reported significant differences in hypothalamic–pituitary–adrenal axis function, structural and functional brain imaging, and inflammatory markers between MDD subgroups with and without ADS (10). However, our research on the pathophysiological mechanisms of ADS is insufficient, especially the research on the special group of adolescents. We need to dig deeper into the characteristics of ADS and propose potential mechanisms for the poor prognosis associated with ADS as its presence identifies a subpopulation with greater illness-associated burden and hazards.

Attentional bias (AB) refers to selective attention when receiving external information due to an increased sensitivity to specific types of stimuli/information (11). Some studies have found that patients with MDD exhibit attentional bias toward negative information, which manifests as prioritizing negative stimuli and avoiding the allocation of attention to positive stimuli (12–14). In addition to an initial attention bias, a research has revealed that individuals with depression exhibit a negative bias in the processing and memory of information (15). Specifically, compared to their healthy counterparts, patients with depressive disorders demonstrate a heightened tendency to quickly process and sustain negative information, while experiencing difficulty in effectively utilizing positive memories to regulate feelings of sadness (16). Therefore, some scholars have proposed a hypothesis that selective attention to negative information may be a significant contributing factor in the onset, persistence, and development of depressive symptoms (17–19). Based on this hypothesis, researchers have targeted altering initial attentional bias as a key focus in the treatment of depressive disorders and have developed various attentional bias modification training techniques. However, the intervention effects have proven to be unclear (19, 20). This situation compels us to reevaluate the attentional bias characteristics of individuals with depressive disorders.

Indeed, while some researchers support the notion that negative attentional bias is a characteristic of individuals with depressive disorders, there have always been dissenting voices in the field. In one dot-detection task (21), simultaneously presented emotional and neutral faces, reporting no differences between the depression and healthy control groups in the early course of attentional allocation, as well as in the attentional bias in response to sad, happy, and angry faces. Cheng et al. (22) conducted five independent experiments using task paradigms capable of triggering attentional bias in participants with depression, including selective attention, attentional switching, and attentional suppression tasks. Patients with MDD performed similarly to healthy controls, leading the authors to conclude that depression is not characterized by biased attentional processes. Another group also found no direct correlation between negative attentional bias and depressive symptoms (23). Harrison and Gibb (24) conducted a study using eye-tracking technology to investigate attentional bias characteristics in children with depression (with an average age of 11.21 years). The results revealed that children with depressive disorders exhibited attentional avoidance of sad facial stimuli while showing a preference for attending to happy faces. Therefore, there is significant disagreement among current research findings regarding attentional bias characteristics in patients with Major Depressive Disorder, particularly in underage patients. This highlights the need for further and more in-depth research in this area.

Among studies examining relationships between affective disorders and attentional bias, research on anxiety disorders is consistent. Adults with anxiety disorders have shown selective attention to threatening stimuli (25). Zhang et al. (26) found patients with anxiety disorder had attentional bias at the early stage. Kim et al. (27) found that individuals with social anxiety disorder exhibit persistent attentional bias to task-irrelevant social threats. They also proposed that the underlying mechanism for this attentional bias involves excessive activation of the amygdala and sustained activity in the bottom-up attentional networks. A systematic review of studies on attentional bias in children with anxiety disorder (28) reported that, among 4,221 participants (anxiety, *n* = 2,222), those with anxiety disorder had attentional bias values similar to those observed in adults when presented with threatening stimuli, albeit to a lesser extent.

In brief, anxiety is characterized by attentional bias to threat, but findings are inconsistent for depression. As we all know, MDD and anxiety often co-occur in children and adolescents (29). The presence of anxiety distress is likely to have an impact on attentional bias characteristics in individuals with MDD. Previous literature provides inconsistent evidence regarding the attentional bias in depression, and unexamined co-occurring anxiety distress may contribute to this inconsistency. Therefore, when studying attentional bias characteristics in MDD, it is essential to differentiate whether co-occurring anxiety distress is present. Our research aims to assess the role of anxiety distress in attentional bias among adolescents with MDD. If adolescents with MDD exhibit different attentional bias characteristics based on the presence or absence of anxiety distress, we can develop customized attention bias modification interventions targeting these subgroups, thus contributing to a more personalized and tailored treatment approach for adolescents with MDD.

## 2 Materials and methods

### 2.1 Patients

Using the sequential enrollment method, we recruited 97 adolescent patients with MDD who had been hospitalized in the Department of Children and Adolescents at Anhui Mental Health Center between September 2021 and April 2022. The inclusion criteria were: 1) meeting the diagnostic criteria for MDD as listed in the DSM-V; age 13 to 18 years; right-handed; and no obvious impairments in vision or hearing.

We excluded patients with neurological disease, serious physical disease, psychoactive substance abuse, intellectual disability, or an inability to complete the experimental tasks.

A Hamilton Anxiety Scale (HAMA) (30, 31) score greater than or equal to 14 was used to divide adolescents with MDD into MDD/ADS+ (*n* = 50) and MDD/ADS- subgroups (*n* = 47). MDD/ADS+ stands for MDD adolescents with anxiety distress; MDD/ADS- stands for MDD adolescents without anxiety distress.

A total of 48 healthy adolescents matched to the MDD group for age, sex, and years of education were recruited from two general middle schools in Hefei City, Anhui Province. They voluntarily participated in the study, with the same exclusion criteria as in the patient group. The general characteristics of each group are shown in **Table 1**.

**Table 1:** Characteristics of study participants.

	MDD/ADS+	MDD/ADS-	HC	Metrics
*n*	50	47	48	
Sex (M:F)	17:33	18:29	19:29	*χ* ^2^(2) = 0.360*, P* = 0.835
Age	14.78 ± 1.52	15.21 ± 1.43	14.71 ± 1.49	*F* = 1.619*, P* = 0.202
Years of Education	9.74 ± 1.67	10.36 ± 1.51	9.73 ± 1.75	*F* = 2.310*, P* = 0.103
HAMA	22.52 ± 5.90	8.23 ± 3.28	0.00 ± 0.00	*F* = 411.899*, P* = 0.000

MDD/ADS+, MDD adolescents with anxiety distress; MDD/ADS-, MDD adolescents without anxiety distress; HC, healthy control group; HAMA, Hamilton Anxiety Scale.

### 2.2 Informed consent and confidentiality

The study protocol was reviewed and approved by the Ethics Committee of Anhui Mental Health Center. All participants and/or their guardians were conscious of the content and purpose of the study. All agreed to and signed the informed consent form. We confirmed that all methods were performed in accordance with the relevant guidelines and regulations.

### 2.3 Methods

The two psychiatrists who assessed the participants were professionally trained and qualified to rate the neuropsychological tests and related scales, with guidance and training provided by the Cognitive Psychology Laboratory jointly established by the Anhui Mental Health Center and Anhui Medical University. Each participant completed the Hamilton Anxiety Scale and attentional bias tests described below. To minimize the interference of hospitalization duration on the study results, all assessments were conducted within one week after patients’ admission. In order to ensure consistency, the assessments for both patients and healthy adolescents were carried out by two trained psychiatrists, who were not the patients’ treating clinicians.

#### 2.3.1 Hamilton anxiety scale

This test includes 14 items. The total score is designed to reflect the severity of anxiety symptoms with reliability and validity.

#### 2.3.2 Attentional bias test

A point-detection paradigm was used with reference to the attentional bias measurement program written by Prof. Xuemin Zhang’s team at the School of Psychology, Beijing Normal University, written using E-Prime 3.0 experimental software (32). The attentional bias determination task was comprised of four steps:

- A “+” gaze point appeared at the center of the computer screen for 500 ms.
- A face picture (neutral or negative) appeared on each side of the gaze point for 500 ms. The pictures were 16 selected from the Chinese Affective Face Picture System (33); half were negative and half were neutral. Half were male, half were female.
- The response target was a capital letter “E” or “F” displayed on the screen. After the pictures disappeared, the response target appeared on either the left or right side of the gaze point for 500 ms, at one of the positions in the previous pictures. At the same time, the participants were asked to put their left index finger on the “E” key and their right index finger on the “F” key and to ensure that neither finger left the keyboard during the experiment.
- When the target appeared, participants were instructed to press the corresponding key on the keyboard as quickly and accurately as possible, with a maximum threshold time of 2,000 ms.
- The center of the screen then turned blank for 1,000 ms, indicating that participants should prepare for the next round.

The attentional bias task consisted of 8 practice sessions and 128 test sessions, which took approximately 8 minutes to complete the process. The computer recorded each response. At the end of the assessment, we calculated average response time (RT), correct response rate (CRR), and attentional bias values (AB value) for the negative and neutral pictures. The attentional bias value was calculated by subtracting the average RT for neutral pictures from that for negative pictures. Larger attentional bias values indicated a more significant attentional bias.

### 2.4 Statistical methods

Data were analyzed using SPSS 24.0 (34). Comparisons among the three groups were performed using the *χ* ^2^ test for sex and analysis of variance (ANOVA) for age, years of education, and HAMA scores. RT, CRR, and AB values did not conform to normal distributions using the Shapiro-Wilk test (35), the parameters of which are provided in the **Appendix**. Therefore, we used nonparametric tests, using the median for statistical descriptions.

To evaluate the difference of single metric (*i.e.* RT, CRR and AB values) between the three groups, we implement the Kruskal-Wallis test. Additionally, the Wilcoxon signed-rank test was conducted intra-group to compare the RT and CRR for the negative and neutral pictures. Spearman correlation analysis was also performed. Differences with *P <* 0.05 were considered statistically significant.

## 3 Results

### 3.1 Comparisons of RT, CRR, and AB values

**Figure 1:**
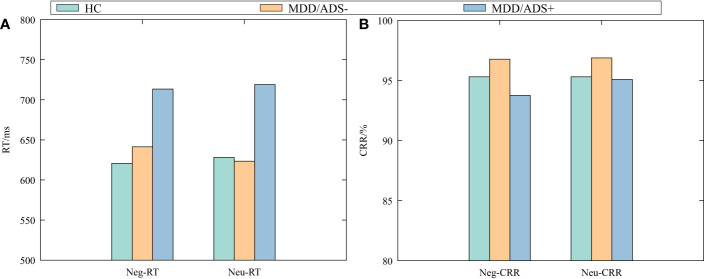
Negative and neutral response times (RT) **(A)** and correct response rates (CRR) **(B)** by group.

**Table 2** shows the results of the multiple independent samples Kruskal–Wallis test for the five indicators in each group: Neg-RT, Neu-RT, Neg-CRR, Neu-CRR, and AB value. Significant differences among groups were observed for Neg-RT, Neu-RT, and Neg-CRR (*P <* 0.05) but not for AB value or Neu-CRR (*P >* 0.05). Dunn’s test was used to further compare pairs with differences (**Table 3**). Neg-RT and Neu-RT were significantly higher in the MDD/ADS+ group than in the MDD/ADS- and HC groups (*P <* 0.01), while Neg-CRR was significantly lower in the MDD/ADS+ group than in the MDD/ADS-group (*P <* 0.05). **Figure 1** presents a more visual comparison of RT and CRR among the three groups.

**Table 2:** Kruskal-Wallis testing for three independent samples.

Metrics	Neg-RT	Neu-RT	Neg-CRR	Neu-CRR	AB value
*H*	20.961	21.428	8.495	3.366	4.165
*df*	2	2	2	2	2
*P*	0.000	0.000	0.014	0.186	0.125

**Table 3:** Pairwise comparison of Neg-RT, Neu-RT and Neg-CRR using Dunn’s Test.

	Group A	Group B	Median A	Median B	Diff (A-B)	*H*	*P*
Neg-RT (ms)	HC HC MDD/ADS-	MDD/ADS- MDD/ADS+ MDD/ADS+	620.73 620.73 641.33	641.33 713.48 713.48	-20.60 -92.75 -72.15	-1.123 -4.402 -3.244	0.784 0.000 0.004
Neu-RT (ms)	HC HC MDD/ADS-	MDD/ADS- MDD/ADS+ MDD/ADS+	628.09 628.09 623.39	623.39 719.07 719.07	4.7 -90.98 -95.68	-0.472 -4.219 -3.719	1.000 0.000 0.001
Neg-CRR	HC HC MDD/ADS-	MDD/ADS- MDD/ADS+ MDD/ADS+	95.31% 95.31% 96.77%	96.77% 93.75% 93.75%	-1.46% 1.56% 3.02%	1.154 2.900 -1.734	0.249 0.745 0.011

### 3.2 Inter-group RT and CRR comparisons between negative and neutral stimuli

**Table 4** shows the results of the Wilcoxon signed-rank test, which was conducted intra-group to compare the RT and CRR for the negative and neutral pictures. Differences in Neg-RT and Neu-RT, Neg-CRR and Neu-CRR within the three groups were not statistically significant (*P >* 0.05).

**Table 4:** Wilcoxon signed-rank test for within-group comparisons.

	Neu-RT − Neg-RT	Neu-CRR − Neg-CRR
HC	*Z* = −1.159*, P*= 0.246	*Z* = −0.082*, P*= 0.935
MDD/ADS-	*Z* = −1.619*, P*= 0.105	*Z* = −0.289*, P*= 0.773
MDD/ADS+	*Z* = −0.594*, P*= 0.553	*Z* = −1.391*, P*= 0.164

### 3.3 Correlation between AB and HAMA scores in adolescents with MDD

Spearman’s correlation analysis showed that HAMA scores in the MDD group were positively correlated with Neg-RT and Neu-RT (*r* = 0.280, *P* = 0.005; *r* = 0.319, *P* = 0.001), negatively correlated with Neg-CRR and Neu-CRR (*r* = −0.285, *P* = 0.005; *r* = −0.228, *P* = 0.025), and not significantly correlated with AB value (*P* = 0.108 *>* 0.05).

## 4 Discussions

To clarify the role of anxiety distress on the attentional bias of MDD adolescents, we compared the attentional bias characteristics of the MDD/ADS+ group, MDD/ADS- group, and healthy control group. The findings indicate that: adolescents with MDD did not show a negative attentional bias when exposed to negative and neutral stimuli, as evidenced by similar reaction times. Compared to the MDD/ADS-group, the MDD/ADS+ group exhibited longer reaction times for negative and neutral stimuli (Neg-RT, Neu-RT), as well as lower correct response rates for negative stimuli (Neg-CRR). Additionally, there were positive correlations between Neg-RT and Neu-RT with HAMA scores, and negative correlations between Neg-CRR and Neu-CRR with HAMA scores among adolescents with MDD.

Attention includes multiple components, including orienting, maintenance, distraction, and conversion (36). In attentional bias research, we consider attention as a cognitive process that enables the parsimonious and efficient allocation of neuronal processing resources (37). These processes are characterized by their limited capacity and selectivity in terms of which stimuli or features are prioritized and focused on (38, 39). The selection processes can be influenced by both neural mechanisms of top-down attentional control (40) and the inherent stimulus information (41). For humans, facial expressions are prominent nonverbal means of expressing and communicating emotional states (42, 43). People naturally focus on emotional faces. So we use emotional faces as stimulus information in our attentional bias task. Theoretically, the attentional bias effect would be enhanced using emotional faces as stimuli in the attentional bias task. However, we found no statistically significant differences among the groups between negative and neutral reactions or between negative and neutral accuracy, suggesting no attentional bias towards negative stimuli in MDD adolescents or healthy adolescents. This finding seems to be different from the results of many previous studies, and it illustrates that the bias toward negative emotional information is not a characteristic of the attentional processes of MDD adolescents. The point-detection paradigm is an indirect assessment of attentional processes while eye-tracking technology can directly and continuously measure the attentional processing of emotional stimuli by recording eye movements. Our conclusion is consistent with recent eye-tracking studies on attentional bias in patients with depression, which detected no significant group differences in the initial attention orientations to sad, happy, and angry faces between patients with depression and healthy individuals (15, 44, 45). The varying results obtained from attentional bias studies conducted in individuals with MDD may be explained by the model of Mogg et al. (46), in which all individuals selectively focus on stimuli that are perceived as dangerous, with differences in attentional responses primarily governed by their subjective evaluations of environmental stimuli, meaning that the threshold for perceiving dangerous stimuli directly affects whether attentional bias is observed. Indeed, individuals who exhibit negative attentional bias tend to have a lower threshold for perceiving stimuli as threatening. However, further research is needed to understand how affective disorders specifically influence such thresholds.

RT for targets on the same side as negative stimuli were significantly longer in the MDD/ADS+ group than in the MDD/ADS- and HC groups. In the point-detection paradigm, there are two explanations for the prolonged reaction time to negative stimuli. The first is that participants have difficulty disengaging their attention from negative stimuli. The second is that participants avoid negative stimuli when initially orienting attention. The first explanation is consistent with the observations of Yiend and Mathews (25): anxiety-related attentional bias are characterized by specific difficulties directing attention away from the location of any threat. In our study, we found that MDD adolescents with anxiety distress have specific difficulties directing attention away from negative emotional information. The second explanation is consistent with the observations of Price et al. (47): adolescents with anxiety disorder strategically avoid negative or threatening stimuli. MacLeod and Grafton (48) suggested that the mechanism of anxiety may undergo longitudinal changes with age, so attentional bias towards negative or threatening stimuli may be an important factor in the onset and early maintenance of anxiety. However, it may gradually be replaced during disease progression by compensatory or secondary mechanisms, maintaining anxiety and habitual avoidance of negative or threatening stimuli. The vigilance-avoidance hypothesis (49) also proposes that anxiety-related attentional bias changes over time. Initially, individuals maintain vigilance towards negative or threatening stimuli and then subsequently engage in avoidance behaviors. In the current study, MDD adolescents with anxiety distress had significantly lower CRR to negative faces than did those without anxiety distress and HC, suggesting that the MDD/ADS+ group did not use prolonged response times to improve accuracy, but more likely avoided negative/threatening stimuli. Moreover, we observed a positive correlation between RT and HAMA scores in adolescents with MDD, as well as a negative correlation between CRR and HAMA scores, suggesting that with more severe anxiety, avoidance behaviors related to anxiety were increased.

This study had some limitations. First, it was a cross-sectional study, and the cross-sectional studies cannot assess changes in attentional bias over time and are also unable to establish causal attribution. Second, one limitation of this study is the relatively small sample size. Third, positive emotional faces were not included in our paradigm.

In summary, our study revealed that negative attentional bias is not a stable cognitive trait in adolescents with MDD, and avoidance or difficulty in disengaging attention from negative emotional stimuli may be the attentional bias characteristic of MDD adolescents with anxiety distress. However, the current research cannot differentiate between these two aspects. In future studies, eye-tracking techniques can be employed to validate these findings. In clinical practice, attention bias modification is considered a promising approach for treating depression. Importantly, the effectiveness of attention bias modification relies on understanding the attentional bias specific to different subtypes of depression. Our study identified attentional bias characteristics in adolescents with comorbid anxiety distress, providing guidance for improving attention bias modification protocols in the future, thus achieving more precise and personalized medical interventions.

## Data availability statement

The raw data supporting the conclusions of this article will be made available by the authors, without undue reservation.

## Ethics statement

The studies involving humans were approved by Ethics Committee of the Affiliated Psychological Hospital of Anhui Medical University. The studies were conducted in accordance with the local legislation and institutional requirements. Written informed consent for participation in this study was provided by the participants’ legal guardians/next of kin. Written informed consent was obtained from the minor(s)’ legal guardian/next of kin for the publication of any potentially identifiable images or data included in this article.

## Author contributions

RY: Conceptualization, Data curation, Methodology, Writing – original draft, Writing – review & editing. HYZ: Project administration, Writing – review & editing. XC: Investigation, Writing – review & editing. DM: Investigation, Writing – review & editing. ML: Data curation, Writing – review & editing. WL: Data curation, Writing – review & editing. HZ: Funding acquisition, Project administration, Writing – review & editing.

## Appendix: the shapiro-wilk test results

**Table A1** depicts the results of the Shapiro-Wilk test, as is mentioned in Section 2.4.

**Table A1:** The results of the Shapiro-Wilk test.

		*W*	*df*	*P*
Neg RT	HC MDD/ADS- MDD/ADS+	0.916 0.935 0.841	48 47 50	0.002 0.012 0.000
Neu RT	HC MDD/ADS- MDD/ADS+	0.937 0.895 0.833	48 47 50	0.012 0.000 0.000
Neg CRR	HC MDD/ADS- MDD/ADS+	0.864 0.914 0.899	48 47 50	0.000 0.002 0.000
Neu CRR	HC MDD/ADS- MDD/ADS+	0.818 0.845 0.865	48 47 50	0.000 0.000 0.000
AB value	HC MDD/ADS- MDD/ADS+	0.980 0.987 0.821	48 47 50	0.590 0.890 0.000
